# Retraction: NFIC suppressed the development of Glioma via modulating the balance of SHP2/PI3K and NF-κB/PTEN Signaling

**DOI:** 10.1371/journal.pone.0350255

**Published:** 2026-05-27

**Authors:** The PLOS One Editors

The *PLOS One* Editors retract this article [1] due to concerns about potential manipulation of the publication process. These concerns call into question the validity and provenance of the reported results. We regret that the issues were not identified prior to the article’s publication.

FW did not agree with the retraction. HBao responded but expressed neither agreement nor disagreement with the editorial decision. KL, NG, YS, HBai, CD, and FJ either did not respond directly or could not be reached.
